# Identifying the molecular function of Tulp3 in neural tube closure and patterning

**DOI:** 10.1186/2046-2530-1-S1-P69

**Published:** 2012-11-16

**Authors:** K Hakim-Rad, V Patterson, A Paudyal, J Murdoch

**Affiliations:** 1 Royal Holloway University of London, UK; 2 Mammalian Genetics Unit, MRC Harwell, UK

## 

The ENU mutagenesis-derived *hitchhiker* mouse mutant exhibits exencephaly and spina bifida, as well as abnormal dorso-ventral patterning of the spinal cord and preaxial polydactyly. The phenotype is derived from a single base pair transversion in the *Tubby-like-protein 3* gene (*Tulp3*) resulting in a splicing defect that almost completely inactivates protein production. Loss of Tulp3 function affects patterning through downstream activation of the Shh signaling pathway and an increase in ventral signaling. Tulp3 protein can be detected in the primary cilia, and Tulp3 depletion affects ciliary accumulation of some G-protein coupled receptors. We have identified Tulp3 interacting proteins using a yeast 2-hybrid approach. These Tulp3-associated proteins include Rho-guanine nucleotide exchange factor, Rgnef and an E3 ubiguitin ligase, Trim71. We show both Rgnef and Trim71 are expressed within the neural tube at the time of neural tube closure and patterning. We have confirmed the interactions with Tulp3 by co-immunoprecipitation in cell culture, and we have preliminary data that demonstrate ubiquitination of Tulp3 in vitro. Furthermore, through use of a cell-based assay, preliminary results show Trim71 knock-down results in Shh signaling pathway suppression. We propose Trim71 and Rgnef provide important mediators in effecting the functional role of Tulp3.

